# The Long and the Short of It: *NEAT1* and Cancer Cell Metabolism

**DOI:** 10.3390/cancers14184388

**Published:** 2022-09-09

**Authors:** Nadine E. Smith, Phaedra Spencer-Merris, Archa Hannah Fox, Janni Petersen, Michael Z. Michael

**Affiliations:** 1 Flinders Health and Medical Research Institute, Cancer Program, Flinders University, Bedford Park, SA 5042, Australia; 2 School of Human Sciences, School of Molecular Sciences, The University of Western Australia, Crawley, WA 6009, Australia; 3 Department of Gastroenterology and Hepatology, Flinders Centre for Innovation in Cancer, Flinders Medical Centre, Bedford Park, SA 5042, Australia

**Keywords:** *NEAT1_1*, *NEAT1_2*, metabolism, paraspeckle, cancer

## Abstract

**Simple Summary:**

Altered metabolism is a hallmark of most cancers. The way that cancer cells regulate their energy production to fuel constant proliferation has been of interest with the hope that it may be exploited therapeutically. The long noncoding RNA, *NEAT1*, is often dysregulated in tumours. *NEAT1* RNA can be transcribed as two isoforms with different lengths, with each variant responsible for different functions. This review explores how the isoforms contribute to cancer metabolism.

**Abstract:**

The long noncoding RNA *NEAT1* is known to be heavily dysregulated in many cancers. A single exon gene produces two isoforms, *NEAT1_1* and *NEAT1_2,* through alternative 3′-end processing. As the longer isoform, *NEAT1_2* is an essential scaffold for nuclear paraspeckle formation. It was previously thought that the short *NEAT1_1* isoform only exists to keep the *NEAT1* locus active for rapid paraspeckle formation. However, a recent glycolysis-enhancing function for *NEAT1_1*, contributing to cancer cell proliferation and the Warburg effect, has been demonstrated. Previous studies have mainly focused on quantifying total *NEAT1* and *NEAT1_2* expression levels. However, in light of the *NEAT1_1* role in cancer cell metabolism, the contribution from specific *NEAT1* isoforms is no longer clear. Here, the roles of *NEAT1_1* and *NEAT1_2* in metabolism and cancer progression are discussed.

## 1. Background

In healthy cells, glucose uptake stimulates cell growth through the activation of intracellular signalling pathways, including glycolysis [1]. In cancer, genetic and epigenetic alterations switch these pathways to a permanently “on” state, promoting continuous growth, which depletes key metabolites. As tumour size increases, oxygen availability decreases, limiting the oxygen-dependent final step of the electron transport chain (ETC). This shift from oxidative phosphorylation (OXPHOS) to aerobic glycolysis is termed the Warburg effect [2,3,4]. Although there is an increase in both glycolytic enzymes and activity in many cancers, the Warburg effect alone does not explain why cancer cells maintain enhanced glycolysis in normoxic cell culture nor in circulating blood [4].

**Figure 1:**
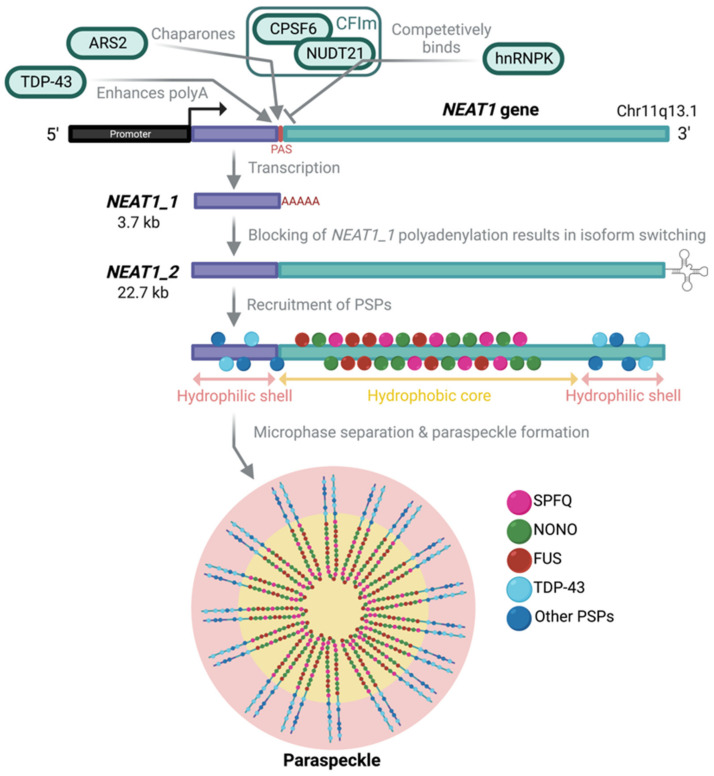
The *NEAT1* gene gives rise to two isoforms with identical 5′ sequences. The paraspeckle-independent *NEAT1_1* undergoes canonical 3′ polyadenylation whilst the blocking of 3′ polyadenylation via competitive binding of hnRNPK to the CFIm complex yields the paraspeckle-essential *NEAT1_2*. The recruitment of paraspeckle proteins to the hydrophilic and hydrophobic regions of *NEAT1_2* leads to phase-separated paraspeckles.

In general, the functions of long noncoding RNAs (lncRNAs) are not well-understood due to their generally low expression levels and high tissue specificity [5]. An exception to this is nuclear enriched abundant transcript 1 (*NEAT1*) dysregulation in many diseases [6]. The highly conserved, single exon, intergenic lncRNA is transcribed near the multiple endocrine neoplasia locus on human chromosome 11q13.1 [7]. Altered 3′-end processing results in two transcripts: 3.7 kb *NEAT1_1* and 22.7 kb *NEAT1_2*; the latter is a well-documented and essential architectural scaffold for subnuclear paraspeckles (see review by McCluggage and Fox, 2021) (Figure 1) [8,9]. A triple-helix structure, processed by RNase P at the 3′-end of *NEAT1_2*, stabilises the transcript to protect it from degradation [9,10]. Less studied is the shorter, polyadenylated, paraspeckle-independent *NEAT1_1* isoform, whose function, until recently, remained elusive. As previously reported, abnormal *NEAT1* expression is correlated with several malignancies, and the distinction between the two isoforms has often been overlooked [11]. Hence, the relative contribution of each isoform to metabolic homeostasis, or pathology, requires attention.

## 2. *NEAT1_1* Enhances the Warburg Effect by Accelerating Glycolytic Metabolite Flux

**Figure 2:**
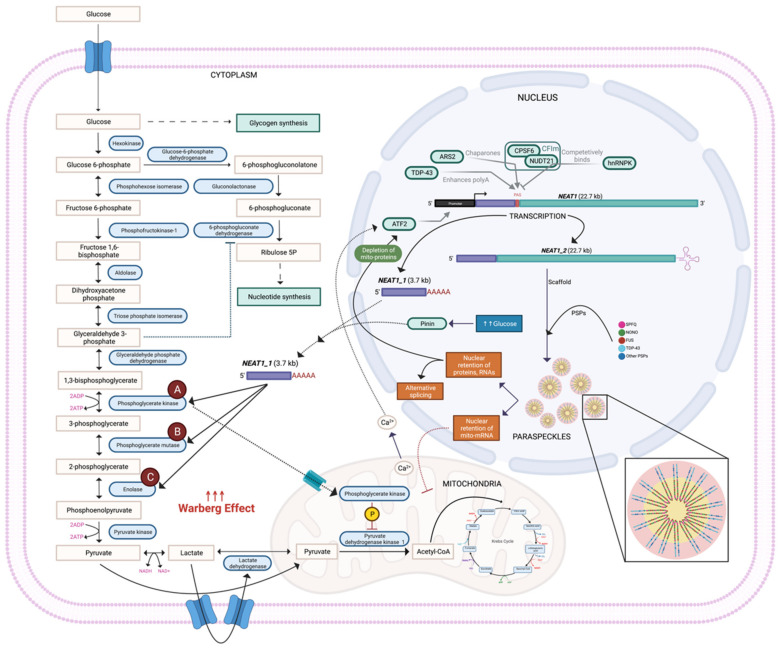
Nucleocytoplasmic transport of *NEAT1_1* enhances the Warburg effect via the binding to glycolytic enzymes. TDP-43, ARS2, and CFIm promote the canonical 3′ polyadenylation of *NEAT1_1,* which can then bind to pinin for nuclear export to the cytoplasm. Once in the cytoplasm, *NEAT1_1* can bind with the glycolytic enzymes PGK1 (**A**), PGAM1 (**B**), and ENO1 (**C**) to enhance glycolytic flux, and hence the Warberg effect, in transformed cells.

Although both isoforms are abundant in the nucleus, *NEAT1_1* is also exported to the cytoplasm [12]. In 2019, Adriaens et al. suggested that *NEAT1_1* is potentially nonfunctional and serves to only keep the transcriptional locus active, making the switch to *NEAT1_2* rapidly available during stress induction [13]. However, a recent study has discovered a novel mechanism of action for *NEAT1_1* in the cytoplasm, in both in vitro and in vivo breast cancer (BC) models [12]. The authors demonstrated that the translocation of *NEAT1_1* from the nucleus to the cytoplasm, through binding with the nuclear speckle-associated protein pinin (encoded by the PNN gene), occurs in a glucose-dependent manner [12,14] (Figure 2). Interestingly, the depletion of pinin reduces cytoplasmic *NEAT1_1* even after glucose stimulation, indicating the importance of pinin in the nucleocytoplasmic transport of *NEAT1_1* [12]. Importantly, in the cytoplasm, *NEAT1_1* interacts with the glycolytic enzymes phosphoglycerate kinase (PGK1), phosphoglycerate mutase (PGAM1), and alpha enolase (ENO1) to promote glycolysis, enhancing growth, proliferation, invasion, and metastasis (Figure 2) [12,15].

PGK1 is constitutively expressed in all somatic and premeiotic cells and is essential in the glycolysis pathway, but it is implicated in cancer as it encompasses many characteristics of an oncogene [16]. This bilobed enzyme has both nucleotide-binding and catalytic domains and is involved in the conversion of 1,3-bisphosphoglycerate (1,3-BPG) to 3-phosphoglycerate (3-PG) in glycolysis (Figure 2A). It also catalyses the first ATP of anerobic glycolysis [16,17]. This substrate-level phosphorylation is of great significance in the continuous production of cellular energy under hypoxic conditions [16]. PGK1 also acts as a protein kinase after translocation to the mitochondria, where it directly phosphorylates pyruvate dehydrogenase kinase isozyme 1 (PDHK1) [17]. Phosphorylated PDHK enhances pyruvate dehydrogenase E1α, which inactivates pyruvate dehydrogenase, preventing the conversion of pyruvate to coenzyme A (CoA), thus suppressing mitochondrial pyruvate metabolism and increasing lactate production [18]. This rate-limiting enzyme plays a role in the promotion of tumourigenesis through the activation of oncogenic pathways, such as Akt/mTOR, Myc, and ß-catenin, and post-translational modifications, such as phosphorylation, acetylation, ubiquitination, and succinylation (as reviewed by Liu et al., 2022) [19]. In vitro research by Gou et al. [20] and Wang et al. [21] have shown that the small-molecule inhibitor of PGK1, NG52, had a dose-dependent effect on the proliferation of ovarian cancer and glioma cells, respectively.

PGAM1 is a highly conserved glycolytic isomerase enzyme involved in the conversion of 3-PG to 2-phosphoglycerate (2-PG), whilst also supporting antioxidative defences by reducing mitochondrial reactive oxygen species (ROS) (Figure 2B) [22,23]. Several studies have linked PGAM1 to cancer progression. Earlier works report *PGAM1* knockdown by short hairpin RNA (shRNA) results in an increase in 3-PG and a subsequent decrease in 2-PG, whilst also decreasing glycolysis, pentose phosphate pathway flux, biosynthesis, and cell proliferation in diverse solid and leukaemia cell lines [24]. Investigation of dysregulated PGAM1 levels in human urothelial bladder cancer tissues found a positive correlation with histological-grade tumours, when compared to adjacent normal tissue [25]. Loss of functional tumour-suppressor protein p53, encoded by tumour protein 53 (TP53), is common in cancer, and it has been found to upregulate both *NEAT1* and *PGAM1* [23]. Similar to many metabolic enzymes, PGAM1 asserts a nonenzymatic function, as the physical interaction with checkpoint kinase 1 (Chk1) increases proliferation, specifically in RAS-driven cancer cells [26].

Alpha enolase (ENO1), encoded by *ENO1*, is another tumour-related, multifunctional protein, which is responsible for the conversion of 2-PG to phosphoenolpyruvate (PEP) in glycolysis (Figure 2C). An increase in *ENO1* expression has been reported in human diseases (i.e., systemic sclerosis, type II diabetes mellitus, lupus, Alzheimer disease) and many cancers, as well as being involved in cell growth and hypoxia tolerance [27,28,29,30,31,32,33,34]. Additionally, silencing *ENO1* reduces the rate of glycolysis in cell lines, favouring OXPHOS even when glucose influx remains high [35]. *ENO1* expression is correlated with colorectal cancer (CRC) progression, and the newly identified protein translational modification, lysine crotonylation, has been identified at lysine residue 420 in CRC cell lines [33]. Interestingly, although *ENO1* is reportedly overexpressed in many human diseases and cancers, in non-small-cell lung cancer (NSCLC), *ENO1* is downregulated at the protein level even though its mRNA levels remain elevated, suggesting post-transcriptional regulation [36,37].

Given the cancer-specific roles for each of these enzymes, the role that *NEAT1_1* plays, either in glycolysis or in sequestering enzymes from other activities, requires further investigation.

## 3. *NEAT1_2* and Paraspeckle Abundance Increase following Stress

First described in 2002 by Fox et al., paraspeckles are discrete, subnuclear bodies, measuring approximately 360 nm in diameter [38,39,40]. Paraspeckle formation relies solely on the generation of *NEAT1_2* in the nucleus, which sets them apart from cytoplasmic stress granules and P bodies, which require multiple proteins and RNA elements to form [8]. Paraspeckle formation occurs only following the recruitment of proteins, such as non-POU-domain-containing octamer-binding protein (NONO), splicing factor proline and glutamine-rich (SFPQ) and fused in sarcoma (FUS) proteins, among others, to the mid-region of *NEAT1_2* transcript. Once localised, the high concentration of molecules aggregates to form a distinct spheroid with spatial organisation [41] (Figure 1). Hydrophilic proteins bind 3′ and 5′ regions of the *NEAT1_2* transcript to form the paraspeckle shell, whilst the middle segment of the transcript forms the hydrophobic core [8,10,38,42]. Individual paraspeckles are spheroidal, but during stress conditions, they can be linked together to generate elongated paraspeckle structures [15]. In HeLa cells, there are ~5–20 paraspeckles per nucleus [43]. However, in 2020, Grosch et al. reported that the size of the nuclei likely influences paraspeckle abundance in human pluripotent stem cells [43]. Regardless of basal paraspeckle abundance, their numbers seem to increase during stress, suggesting that *NEAT1_2* accumulates.

## 4. What Is Driving the *NEAT1* Isoform Switch?

Since the formation of paraspeckles is dependent on the transcriptional read-through of *NEAT1_1* to *NEAT1_2*, understanding the mechanistic control of this isoform switch is crucial. Polyadenylation (polyA) signals terminate the primary *NEAT1* transcript, resulting in canonical processing of the 3′ polyA tail and consequently a short *NEAT1_1* lncRNA [44]. The long *NEAT1_2* isoform is generated when heterogeneous nuclear ribonucleoprotein K (hnRNPK) competes with cleavage-and-polyadenylation-specific factor 6 (CPSF6) for nudix hydrolase 21 (NUDT21) binding, inhibiting the CPSF6–NUDT21 (CFIm) complex from forming, and facilitating the 3′-end polyA (Figure 2) [44,45]. In vitro binding assays have demonstrated that inhibiting the formation of the CFIm complex prevents the polyadenylation of *NEAT1_1* and increases the nuclear levels of *NEAT1_2* and, thereby, paraspeckles [44,46]. Additionally, recent work has reported that arsenic resistance protein 2 (ARS2) acts as a chaperone, guiding CFIm to the *NEAT1* transcript to facilitate the polyadenylation of *NEAT1_1* in osteosarcoma cell lines [47]. Furthermore, the knockdown of ARS2 leads to an increase in, and the preferential stabilisation of, *NEAT1_2* transcripts [47]. Moreover, RNA binding protein (RBP) transactive response (TAR) DNA binding protein 43 kDa (TDP-43) directly represses the formation of paraspeckles, but it increases *NEAT1_1* transcription by binding the *NEAT1_1* GU-rich motifs upstream of the polyA site [48,49]. Although TDP-43 has been thoroughly investigated in amyotrophic lateral sclerosis and has been linked to altered miRNA expression, the understanding of its involvement in a cancer context remains limited [49,50,51,52,53,54,55]. In summary, the isoform switch from *NEAT1_1* to *NEAT1_2* may involve several factors with context-specific roles; their oncogenic relevance requires further clarification.

## 5. *NEAT1* and Paraspeckles Alter Metabolism via Mitochondria

Mitochondrial function reaches beyond the established role in energy generation. In addition to ATP production, mitochondria generate macromolecules which alleviate mitochondrial stress [56]. Interestingly, the mitochondrial stressors, FCCP, rotenone, and doxycycline, all increase *NEAT1* levels, in part through ATF2-induced *NEAT1* expression [10]. Mito–nuclear communication is crucial for ensuring cellular homeostasis during mitochondrial stress, and recently it has been hypothesised that *NEAT1* and paraspeckles may play a role in mitochondrial homeostasis [57,58]. Mitochondrial fusion and fission are controlled by dynamin-related GTPases MFN1/MFN2 and DRP1, respectively [58]. *NEAT1*-depletion in HeLa and HEK293 cells resulted in mitochondrial elongation and the enhanced retention of mito-mRNAs encoding functional mitochondrial proteins, such as cytochrome c, subunits of NADH dehydrogenases, and carnitine o-palmitoyl transferase [10]. This was further supported by reduced *DRP1* but stable *MFN1* and *MFN2* expression, increased mitochondrial mRNAs (mito-mRNAs) exported from the nucleus, reduced respiration capacity, ATP generation, extracellular acidification rate (ECAR), and proliferation [10]. On the contrary, *NEAT1* overexpression showed an increase in *DRP1* expression and DRP1 phosphorylation, corresponding to fragmented mitochondria and increased mitochondrial DNA (mtDNA), and this was phenocopied by deleting the *NEAT1_1* polyadenylation signal [10].

## 6. Alternative Processing of lncRNAs in Cancer

Many lncRNAs are implicated in cancer, either through direct or indirect processing, as previously reviewed [59,60]. Similarly to *NEAT1*, the noncoding product of a neighbouring locus metastasis-associated lung adenocarcinoma transcript 1 (*MALAT1*) is retained in the nucleus and is involved in the nuclear architecture as well as in RNA splicing and gene-regulation [61]. Whilst *MALAT1* has been found to be overexpressed in 14% of breast tumour samples, an alternatively spliced variant of *MALAT1* (Δsv-*MALAT1*) showed decreased expression in a subset of tumours and shows potential as an individual prognostic factor for BC [62]. Additionally, similar to *NEAT1*, *MALAT1* contains a tRNA-like clover-leaf structure near the 3′ terminus; however, this structure is cleaved [63] and accumulates as *MALAT1-*associated small cytoplasmic RNA (mascRNA) in the cytoplasm, where it promotes global translation [64] and hepatocellular cancer cell proliferation [65]. Additionally, the lncRNA *ANRIL* recruits polycomb proteins to regulate target-gene expression, and the overexpression of truncated isoforms has been reported in bladder cancers [66]. Clearly, the function of many lncRNAs is nuanced and not limited to the full-length transcript. Understanding the activities of truncated forms and processed derivatives of lncRNAs will enlighten our understanding of these complex regulatory molecules.

## 7. Dysregulation of Both Short and Long *NEAT1* in Cancer

*NEAT1* (without distinguishing between the long and short isoform) is expressed at similar levels in most healthy tissues, but it is reported to have a relatively low expression in the brain, heart, and whole blood [6]. On the contrary, *NEAT1* is upregulated in many solid cancers (reviewed in [6,67,68,69,70]), and in most cases, it is associated with aggressive disease and poor outcomes [67,68,69]. However, only a handful of recent studies have established the relative abundance of the two isoforms, despite many reports suggesting that *NEAT1* acts as either a tumour suppressor or oncogene, depending on this isoform ratio (summarised in Table 1) [70,71,72,73,74]. In some haematological malignancies, *NEAT1* functions as a tumour suppressor, enhancing the expression of *NEAT1_2,* and thus paraspeckle formation, which may counteract oncogene-induced stressors in cancer [75,76]. *NEAT1* levels are upregulated in multiple myeloma (MM) when compared to healthy controls; however, no correlation with patient prognosis has been found [77]. In chronic lymphocytic leukaemia (CLL), the total *NEAT1* expression levels remained similar to those of healthy controls, but the expression of *NEAT1_2* was found to be significantly higher [77]. On the other hand, in acute and chronic myeloid leukaemia (AML and CML, respectively) and acute lymphoblastic leukaemia (ALL), total *NEAT1* levels decrease in patients’ peripheral blood and bone marrow, and this was found to be an essential mediator of apoptosis induced by imatinib in BCR-ABL-expressing cells [75,77,78,79,80].

Tumour suppressor p53 is regarded as the guardian of the genome, but it is mutated in over 50% of malignancies, enabling cells to escape apoptotic signalling, bypass cell-cycle arrest, and inhibit senescence [112,113]. It is well-established that *NEAT1* is induced by p53 binding to the *NEAT1* promotor to activate expression [76,112,114]. Interestingly, *NEAT1* also promotes p53 and Chk1 through ATR signalling in response to replication stress [76]. Furthermore, in CRC cells, the induction of both *NEAT1* isoforms were p53-dependent when exposed to a chemotherapeutic agent and the topoisomerase 2 inhibitor, doxorubicin [112]. In CML, p53 mutations are uncommon [78]; instead, MYC binds to the *NEAT1* promotor to enhance expression [78]. Accordingly, Ronchetti et al. reported an increase in total *NEAT1* and *NEAT1_2* expression in CML patients when compared to normal B-cells [77].

A positive correlation was identified for increased *NEAT1* expression and higher histological grades of BC, and *NEAT1* levels were elevated in patient plasma and peripheral blood although no relative abundance of *NEAT1* isoforms was reported [11]. The same study reported higher expression of *NEAT1* in estrogen receptor positive (ER+) breast cancers, when compared to estrogen-receptor-negative- (ER-) BC [11]. Similarly, correlation to lymph node positivity was seen with ER+, but not with ER- [11]. Evidence suggests that *NEAT1* point mutations are drivers for breast and prostate cancers, regardless of little change in *NEAT1* transcription levels [115,116,117]. However, a more recent study suggested these mutations were likely caused by transcription errors instead of cancer-specific selection pressures [118]. Regardless, *NEAT1* downregulation has been reported in invasive breast carcinoma, oesophageal cancer, pheochromocytoma, and paraganglioma, suggesting *NEAT1* plays a tumour-suppressor role in these malignancies [6]. In summary, it is apparent that *NEAT1* expression varies greatly between malignancies and that relative isoform abundance may play a key role in disease progression.

## 8. Considerations for Isoform Detection of *NEAT1*

Most recent *NEAT1* studies have concentrated on the paraspeckle-associated long isoform *NEAT1_2*, at the risk of overlooking essential roles for the shorter *NEAT1_1*, paraspeckle-independent isoform. In addition, an analysis of the literature (Table 1) shows the reliance placed upon glyceraldehyde-3-phosphate dehydrogenase (GAPDH) transcripts levels for normalizing *NEAT1* expression in RT-PCR studies. As new light has been shed on the involvement of *NEAT1_1* in glycolysis, a glycolytic housekeeping transcript, such as *GAPDH,* may not be ideal for normalizing *NEAT1* expression in studies related to metabolism.

Inconsistent polyadenylation and contextual processing can make lncRNAs difficult to quantify using standard RT-PCR protocols. Quantifying the relative abundance of *NEAT1* isoforms is not straightforward. Kolenda et al. [119] compared cDNA synthesis protocols for various cancer-associated lncRNAs; however, isoform differentiation was not a primary outcome. Similarly, the RNA purification method used can significantly influence the relative abundance of isoforms, with a heating step liberating *NEAT1_2* from paraspeckle complexes and increasing yields [120]. While oligo-dT primed cDNA might be used to specifically amplify *NEAT1_1* sequences, the presence of poly-A stretches downstream in the *NEAT1_2* sequence necessitates the careful calibration of reverse transcription conditions, which is rarely reported. Validated oligo-dT clamp cDNA protocols would be advantageous.

It may be expected that RNA-seq should enable accurate comparison of isoforms; however, many studies report oligo-dT primed libraries, such that the long isoform is under-represented or ignored, and even total RNA-seq data may be influenced by the aforementioned bias in isoform ratios as a result of RNA isolation methods. Newer, long-read direct RNA sequencing methods promise improved qualitative and quantitative data [121].

Visualisation of *NEAT1_2*, employing RNA-fluorescence in situ hybridization, is often used to quantify paraspeckle abundance and can also be directed to detect *NEAT1_1* [13]. Similarly, dCas13 tagging has recently been used to detect both isoforms of *NEAT1* in living cells [122].

## 9. Conclusions and Future Directions

In the context of cancer, *NEAT1* may have either a protective, tumour-suppressive role, or a tumour-promoting oncogenic role, depending upon the type of cancer and, most likely, also upon the specific *NEAT1* isoform expressed. Many previous studies have concentrated on the total *NEAT1* transcription level, rather than on the isoform ratio; hence, isoform-specific contributions are unclear. Improving detection of specific *NEAT1* isoforms is crucial in understanding the tumour-promoting vs. the tumour-protective roles of *NEAT1* in cancer. Future directions will compare the relevant contributions of paraspeckle-mediated sequestration and epigenetic regulation against the glycolytic influence of the shorter isoform on tumour progression. Whether one or both isoforms are found to be necessary for specifically maintaining neoplasia, that will impact their relative value as therapeutic targets.

## Figures and Tables

**Table 1:** Summary of *NEAT1* expression studies, as determined by RT-PCR, in various human cancer subtypes, published between 2014 and 2022.

Cancer Type	*n*/Cell Line/Sample Type	*NEAT1* Isoforms Investigated	Major Findings	RT-PCR Control Gene	Year	Ref
**Multiple** **myeloma (MM)**	*n =* 46 MM, *n =* 14 plasma cell leukaemia (PCL) *n =* 628 from publicly available datasets: #GSE5900 (44 MM, 12 MGUS, 22 healthy donors) #GSE2658 and #GSE24080	Total *NEAT1* and *NEAT1_2* *NEAT1_1* and *NEAT1_2* with RNAseq	RNAseq allowed estimated isoform abundance was based on unambiguously mapped reads. ↑ *NEAT1* in tumour samples when compared to healthy controls 90% of total *NEAT1* was *NEAT1_1.* A negative correlation was found between—4*NEAT1* and UPR. Neither total *NEAT1* nor *NEAT1_2* correlated with overall survival or time-to-next-treatment. *NEAT1* was not found to be differentially expressed in diverse cell types, i.e., primary vs. secondary cell leukaemia.	Undisclosed	2019	[81]
	*n* = 82 blood samples	Total *NEAT1* and *NEAT1_2*	↑*NEAT1_1*, inferred by the difference in Ct value between total *NEAT1* and *NEAT1_2.*	*GAPDH*	2020	[77]
**B-cell acute lymphoblastic leukaemia (ALL)**	*n =* 16 blood samples		↑*NEAT1_1*, inferred by the difference in Ct value between total *NEAT1* and *NEAT1_2*. ↑ *NEAT1_1 and NEAT1_2*.			
**Acute myeloid leukaemia (AML)**	*n =* 20 blood samples		↑*NEAT1_1*, inferred by the difference in Ct value between total *NEAT1* and *NEAT1_2*. ↑ *NEAT1_1* and *NEAT1_2*.			
**Chronic lymphocytic** **leukaemia (CLL)**	*n =* 310 blood samples		↑*NEAT1_1*, inferred by the difference in Ct value between total *NEAT1* and *NEAT1_2.* Stable total *NEAT1* but *NEAT1_2* (40% of total *NEAT1*). ↑ *NEAT1_2* in patients with IGHV gene mutations. ↓ *NEAT1_2* in patients with Trisomy 12.			
	*n =* 72 peripheral blood samples	Total *NEAT1*	p53 binds to the *NEAT1* promotor in CLL and lymphoma. p21 and *NEAT1* expression levels significantly correlated after irradiation. Nutlin-3 induced *NEAT1* expression 2.3-fold in WT p53 primary CLL cells, compared to 1.2-fold in p53 mutant cells.	*Lamin B1*	2015	[82]
**Chronic myeloid leukaemia (CML)**	*n =* 26 peripheral blood samples	Total *NEAT1 and NEAT1_2*	↓ Total *NEAT1* and ↓ *NEAT1_2*. Silencing BCR-ABL expression total *NEAT1* and *NEAT1_2* in CML cell line K562, suggesting *NEAT1* may regulate BCR-ABL mediated pathways. c-myc represses *NEAT1* transcription by binding to promotor.	*ACTB*	2018	[78]
**Acute promyelocytic** **leukaemia (APL)**	*n =* 31 APL and *n =* 12 normal blood samples NB4, NB4-R2, and U937-PR9 cell lines	Total *NEAT1* and *NEAT1_2*	↓ Total *NEAT1* and *NEAT1_2* in APL patient samples when compared to normal granulocytes. *NEAT1* expression is repressed by PML-RARα fusion gene. *NEAT1* expression is involved in the differentiation of APL cells.	*ACTB*	2014	[75]
**Thyroid** **carcinoma (TC)**	*n* = 98 Peripheral blood and thyroid tissue samples (malignant *n* = 52, benign *n* = 46)	*NEAT1_2*	↑*NEAT1_2* in benign vs. malignant thyroid nodules.	*GAPDH*	2020	[83]
	Circulating blood monocytes (CBMs) and tumour-associated macrophages (TAMs) *n =* undisclosed TPC-1 cell line Bone marrow-derived macrophages and macrophages	Undefined	↑*NEAT1* expression in TAMs, compared to CBMs. *NEAT1* is a direct target of miR-214 in TC cell lines. Knockdown of *NEAT1* impairs malignant progression of thyroid papillary carcinoma and tumour growth in vivo.	*GAPDH*	2017	[84]
**Anaplastic** **thyroid carcinoma (ATC)**	*n =* 25 matched samples SW1736 and KAT-18 cell lines	Total *NEAT1*	↑*NEAT1* in ATC tissues and cells exposed to hypoxic conditions.	*GAPDH*	2020	[85]
**Papillary** **thyroid** **carcinoma (PTC)**	*n =* 20 matched samples NPA87, TPC-1, KAT-5, and HT-ori3 (control) cell lines	Total *NEAT1*	↑*NEAT1* expression in patient PTC samples when compared to adjacent normal tissues. ↑ *NEAT1* expression in PTC cell lines compared to control cells.	*GAPDH*	2018	[86]
**Neuroblastoma**	Publicly available datasets (total *n* = 1062): Versteeg (*n* = 88), Kocak (*n* = 476), and SEQC *(n* = 498)	Total *NEAT1* and *NEAT1_2* (RT-PCR and RNA-FISH)	*NEAT1_1* abundance inferred by subtracting *NEAT1_2* levels from total *NEAT1* levels. ↑ *NEAT1_1:NEAT1*_2 in aggressive neuroblastoma. ↑ *NEAT1_2* and ↑ paraspeckles in nonaggressive neuroblastoma.	*RPLP0*	2021	[87]
**Breast cancer (BC)**	MCF-7, MDA-MB-453, MDA-MB-231, SKBR3, and MCF-10A (control) cell lines	Total *NEAT1*	↑*NEAT1* in all cancer cell lines when compared to control cell line. *NEAT1* was negatively correlated with miR-448.	*GAPDH*	2018	[88]
	*n =* 1065 post-data filtering of TCGA (*n* = 526), Oslo2 (*n* = 378), and METABRIC (*n* = 1904) cohorts. BT474, BT549, HCC1569, Hs578T, MDA-MB-231, MDA-MB-468, MCF7, SK-BR-3, and T-47D cell lines *n =* 74 BC biopsies and *n =* 27 non-malignant biopsies	*NEAT1_2* *NEAT1_1* with RNAseq *NEAT1_2* with RNA-FISH	*NEAT1_1* expression level determined from polyA-selected RNAseq data from TCGA cohort. *NEAT1_1* expression is highest in ER-positive luminal A and B breast cancer. ↑ *NEAT1_2* and paraspeckle abundance correlate with high-grade disease (RNA-FISH). ↑ *NEAT1_2* in HER2-enriched and luminal B BC in all three cohorts. *NEAT1_2* is not expressed at RNA-FISH-detectable levels in normal breast tissue.	Geometric mean of *GAPDH, B2M,* and *RPLP0*	2020	[89]
	MCF-7, MDA-MB-231, and MDA-MB-468 cell lines Gene expression data *n* = 2000	*NEAT1_1* and *NEAT1_2*	*NEAT1_1* transcription was analysed by using a polyA primer for cDNA generation before RT-qPCR, using primers targeting total *NEAT1*. *NEAT1_2* transcription was analysed by using random primers for cDNA generation before primers specifically targeting the *NEAT1_2* region of the transcript. ↑ *NEAT1* associated with poor patient prognosis.	*RPL11*	2015	[90]
	MDA-MB-231 and MCF-10A (control) cell lines	*NEAT1_2* with RNA-FISH	↑ Paraspeckle formation in MCF-7 cell lines when compared to MCF-10A cells. ↑ *NEAT1_2* expression after G-quadruplex (G4)-specific stabilization with small molecules. *NEAT1_2* expression could be regulated by a G4s.	*GAPDH* and *ACTB*	2021	[91]
**Osteosarcoma (OS)**	U2OS cell line	Total *NEAT1* and *NEAT1_2* (RT-qPCR and RNA-FISH). *NEAT1_1* in *NEAT1_2* KO cells	*NEAT1* isoform-specific KO cell lines were achieved using CRISPR-Cas9 technologies. *NEAT1_1* levels were unaltered or increased in some *NEAT1_2^−/−^* lines. *NEAT1_1* localises to nuclear speckles, independent of paraspeckles.	*RPLP0*	2017	[92]
	*n* = 47 biopsies and adjacent matched tissues HOS, SaOS2, MG63, U2OS, and hFOB1.19 (control) cell lines	Total *NEAT1*	↑*NEAT1* expression ↑ HIF-1α in MG63 cells, and this *NEAT1*-mediated HIF-1α expression was reversed by miR-186-5p in HOS cells. ↑ *NEAT1* in OS tissues and cell lines. ↑ *NEAT1* associated with advanced clinicopathologic features and poor overall survival. *NEAT1* promotes proliferation, invasion, and EMT in cell lines. *NEAT1* promoted growth in vivo. miR-186-5p is a downstream target of *NEAT1* in osteosarcoma.	*GAPDH*	2019	[93]
	U2OS cell line	Total *NEAT1* and *NEAT1_2*	Total *NEAT1* levels were slightly higher in CBP80-KD and ARS2-KD cells when compared to control KD cells. *NEAT1_2* alone 5-fold in ARS2-KD cells, but not in CBP80- or PHAX-KD cells. ARS2 suppresses the formation of paraspeckles.	*GAPDH*	2020	[47]
**Ovarian cancer (OC)**	*n =* 30 paired tissue samples; CAOV3, ES-2, and IOSE80 (control) cell lines	Total *NEAT1*	↑*NEAT1* in patient samples and OC cell lines. *NEAT1* knockdown with siRNA increased apoptosis and decreased proliferation, colony formation, migration, invasion, and glycolysis.	*GAPDH*	2020	[94]
	ovarian carcinoma patient specimens (*n* = 18 responsive, *n* = 14 resistant) SKOV3 HeyA-8, PTX-resistant, SKOV3/PTX, and HeyA-8/PTX cell lines. *n* = 10 BALB/c athymic mice	Total *NEAT1*	↑*NEAT1* in treatment-resistant patients when compared to treatment-responsive patients. *NEAT1* knockdown enhanced PTX sensitivity in PTX-resistant OC cells. *NEAT1* negatively regulates miR-194 expression. *NEAT1* sponges miR-194, leading to upregulation of ZEB1 expression. *NEAT1* knockdown improved sensitivity to PTX in OC in vivo.	*GAPDH*	2017	[95]
**Prostate cancer (PC)**	LNCaP, DU145, and RWPE-1 (control) cell lines	Total *NEAT1*	↑*NEAT1* in PCa cells. *NEAT1* negatively regulates hsa-miR-218-5p and has-miR-483-3p when compared to normal prostate epithelial cells.	*GAPDH*	2022	[96]
	Explant cultures from primary, patient-derived bone metastatic prostate panel Primary prostate and bone metastatic tissues Patient-derived xenograft TCGA datasets	Total *NEAT1* Total *NEAT1_1* (RNA-FISH)	↑*NEAT1* in prostate cancer when compared to normal tissues (from TCGA datasets). ↑ *NEAT1_1* predicts poor patient prognosis. *NEAT1_1* enhances prostate-patient-derived xenograft growth through the post-transcriptional RNA modification N6-methyladenosine (m6A). m6A level of *NEAT1_1* correlated to prostate cancer progression and bone metastasis, and negatively correlated to patient survival.	*GAPDH*	2020	[97]
**Hepatocellular carcinoma (HCC)**	blood samples (*n =* 36 HCC, *n* = 36 controls)	Total *NEAT1*	↑*NEAT1* in HCC patient samples when compared to healthy controls. miR-129-5p negatively correlated to *NEAT1* levels.	*GAPDH*	2019	[98]
	*n =* 62 matched biopsies MHCC97H, MHCC97L, SMCC7721 and LO2 (control) cell lines	Total *NEAT1*	↑*NEAT1* in HCC tissues compared to adjacent tissues. ↑ *NEAT1* correlated with tumour size and vascular invasion. *NEAT1* knockdown inhibits proliferation, colony formation, and cell invasion in HCC. miR-613 is a target of *NEAT1* in HCC.	*GAPDH*	2017	[99]
	*n =* 28 biopsies and adjacent tissues HepG2, MHCC97L, MHCC97H, and LO2 (control) cell lines	Total *NEAT1*	↑*NEAT1* expression compared to matched tumour samples. Patients with *NEAT1* expression had HIF-2α expression, whilst patients with—*NEAT1* expression (though still significantly higher than matched samples) had ¯ HIF-2α expression.	*GAPDH*	2018	[100]
**Gastric cancer (GC)**	*n =* 140 samples and *n =* 20 adjacent tissues NCI-N87, SGC-7901, MKN-45, AGS, and GES-1 (control) cell lines	Total *NEAT1*	↑*NEAT1* expression in GC cell lines compared to control cell line. *NEAT1* regulates expression of EMT-associated genes in GC cells; ↓ in vimentin and N-cadherin, ↑ in Zo-1 and E-cadherin; suggests KD of *NEAT1* may inhibit EMT.	*GAPDH*	2016	[101]
**Lung adenocarcinoma (LUAD)**	*n* = 124 biopsies and adjacent tissues A549, CL1-0, and BEAS-2B (control) cell lines	Total *NEAT1*	Overexpression rate of *NEAT1* in lung cancer samples was 90.3%. Significant positive correlations found between *NEAT1* and *Oct4* mRNA expression levels. Oct4 directly binds to *NEAT1* promoter. Lung cancer cell lines A549 and CL1-0 transiently overexpressing Oct4 induced *NEAT1* promoter activity.	*GAPDH*	2017	[102]
	A549, H460, H1650, H1975, H1299, and NHBE (control) cell lines TCGA database: *n* = 687	Total *NEAT1*	↑*NEAT1* expression in all cell lines and patient samples when compared to normal tissue and control cell lines. Positive correlation between ATF2 and *NEAT1* expression in LUAD tissues.	*ACTB*	2020	[103]
**Non-small-cell lung cancer (NSCLC)**	A549, H1299, H460, H1975, and BES-2B (control) cell lines	Total *NEAT1*	↑*NEAT1* expression in all carcinoma cell lines when compared to control cell lines. *NEAT1* promotes growth, migration, and invasion of A549 and H460 cells. *NEAT1* directly targets hsa-miR-98-5p, and its expression was significantly downregulated in NSCLC cell lines when compared to normal lung epithelial cell line. MAPK6 is a direct target of hsa-miR-98-5p in NSCLC cells.	*GAPDH*	2019	[104]
**CRC**	*n =* 30 blood samples, *n* = 30 controls; validation in *n* = 100 patients, *n* = 100 controls. *n* = 29 matched tissue samples, *n* = 19*, whole blood and tissue samples.* HCT116 and LOVO cell lines	Total *NEAT1* and *NEAT1_2*	Details as to how *NEAT1_1* expression was measured were not disclosed. ↑ *NEAT1* in whole blood of CRC patients when compared to normal controls. Total *NEAT1* and *NEAT1_2* expression found to be highly accurate in distinguishing CRC patients from normal controls. KD of *NEAT1_1* inhibits proliferation and invasion. KD of *NEAT1_2* promoted growth. *NEAT1* expression was elevated in neutrophils in CRC patients. ↑ *NEAT1_2* correlated with better overall survival.	*ACTB*	2015	[71]
	*n =* 71 tissue samples and *n =* 61 normal tissue samples from publicly available dataset, RKO, CACO2, SW1116, LOVO, SW480, SW620, HT29, and HCT116 cell lines BALB/c nude mice	Total *NEAT1*	↑*NEAT1* associated with poor prognosis in CRC patients. *NEAT1* mediates cell proliferation in vitro and tumorigenicity in vivo. KD of *NEAT1* significantly inhibited flattening and spreading abilities of HCT116 and SW1116 cells, whilst overexpressing *NEAT1* strongly promoted these abilities in HT29 cells. ↑ E-cadherin and—N-cadherin expressed at both mRNA and protein levels in *NEAT1* KD cells. *NEAT1* OE recovered proliferation potential of CRC cell lines which were impaired by simultaneous downregulation of DDX5. DDX5 correlated with *NEAT1* expression in 71 CRC samples.	*GAPDH*	2018	[105]
	*n =* 12 paired patient samples SW480, HT29, and Caco2 cell lines Nude mice (*n =* 5–7 per group)	*NEAT1_2*	↑*NEAT1* in CRC tissues is negatively correlated with miR-193a-3p. *NEAT1* KD ↑ miR-193a-3p expression and attenuates CRC cells. KRAS acts as a target of miR-193a-3p.	*GAPDH*	2019	[106]
	GEO databases GSE20916 and GSE9348 *n =* 100 and adjacent tissue samples SW620, SW480, HCT116, HT29, CaCo-2, LOVO, and Colo205 cell lines	Total *NEAT1*	↑*NEAT1* in tumour tissue, when compared to normal tissue, in both the independent datasets and in the matched tissue samples. *NEAT1* expression correlated with carcinoembryonic antigen (CEA) levels, tumour size, and distant metastasis. ↑ *NEAT1* predicts overall survival in CRC patients. *NEAT1* regulates cell proliferation and invasion through miR-34a.	*GAPDH*	2019	[107]
**Nasopharyngeal carcinoma (NPC)**	*n* = 96 NPC and *n* = 32 nasopharyngeal epilethium tissues	Total *NEAT1*	↑*NEAT1* expression in patient samples when compared to normal tissues. *NEAT1* expression was negatively correlated with overall survival of NPC patients.	*ACTB*	2019	[108]
**Laryngeal** **cancer (LC)**	*n =* 50 paired patient samples TU686, TU177, AMC-HN-8, and 16HBE (control) cell lines	Total *NEAT1*	miR-340-5p OE↓*NEAT1* stability via direct binding and consequently ↓*NEAT1* expression in LC cells. *NEAT1* OE reversed repression of miR-340-5p OE on LC cell proliferation and invasion.	*GAPDH*	2022	[109]
**Laryngeal** **squamous cell cancer (LSCC)**	*n =* 52 paired tissue samples Hep-2 cell line	Total *NEAT1*	↑*NEAT1* expression in LSCC tumour tissues compared to nonneoplastic tissues. *NEAT1* expression correlated with T grade, neck nodal metastasis, and clinical stages of LSCC. *NEAT1* knockdown inhibited the growth of LSCC xenografts in mice. *NEAT1* knockdown induced apoptosis in LSCC cells in vivo.	*ACTB*	2016	[110]
**Oesophageal squamous cell carcinoma (OSCC)**	EC109, EC9706, and HET-1A (control) cell lines	Total *NEAT1*	↑*NEAT1* expression in EC109 and EC9706 cell lines. *NEAT1* functions as an endogenous sponge for miR-129.	*GAPDH*	2017	[111]

## Abbreviations

ALL	Acute lymphoblastic leukaemia
AML	Acute myeloid leukaemia
APL	Acute promyelocytic leukaemia
ATC	Anaplastic thyroid carcinoma
ATR	Ataxia telangiectasia and Rad3-related
BC	Breast cancer
CBMs	Circulating blood monocytes
CC	Cervical cancer
CFIm	CPSF6-NUDT21 complex
Chk1	Checkpoint kinase 1
CLL	Chronic lymphocytic leukaemia
CML	Chronic myeloid leukaemia
CoA	Co-enzyme A
CPSF6	Cleavage and polyadenylation-specific factor 6
CRC	Colorectal cancer
DRP1	Dynamin-related protein 1
ECAR	Extracellular acidification rate
ENO1	Alpha enolase
ETC	Electron transport chain
FCCp	Carbonyl cyanide 4-(trifluoromethoxy)phenylhydrazone
FUS	Fused in sarcoma
GAPDH	Glyceraldehyde 3-phosphate dehydrogenase
GC	Gastric cancer
HCC	Hepatocellular carcinoma
KD	Knockdown
KO	Knockout
LC	Laryngeal cancer
lncRNA	Long noncoding RNA
LSCC	Laryngeal squamous cell cancer
LUAD	Lung adenocarcinoma
mascRNA	*MALAT1*-associated cytoplasmic RNA
MFN1/2	Mitofusion protein
MM	Multiple myeloma
mtDNA	Mitochondrial DNA
NADH	Nicotinamide adenine dinucleotide (NAD) + hydrogen
*NEAT1*	Nuclear enriched abundant transcript 1
NONO	Non-POU-domain-containing octamer-binding protein
NPC	Nasopharyngeal carcinoma
NSCLC	Non-small-cell lung cancer
NUDT21	Nudix hydrolase 21
OC	Ovarian cancer
OE	Over-expression
OSCC	Oesophageal squamous cell carcinoma
OXPHOS	Oxidative phosphorylation
p53	Tumour suppressor protein 53
PC	Prostate cancer
PDHK1	Pyruvate dehydrogenase kinase isozyme 1
PGAM1	Phosphoglycerate mutase 1
PGK1	Phosphoglycerate kinase 1
PTC	Papillary thyroid carcinoma
RAS	Rat sarcoma virus oncogene
RBP	RNA binding protein
ROS	Reactive oxygen species
SFPQ	Splicing factor proline and glutamine-rich
shRNA	Short hairpin RNA
TAMs	Tumour associated macrophages
TAR	Transactive response
TC	Thyroid carcinoma
TDP-43	TAR DNA binding protein 43 kDa
